# Investigation of the serotonin 2C receptor gene in attention deficit hyperactivity disorder in UK samples

**DOI:** 10.1186/1756-0500-2-71

**Published:** 2009-05-05

**Authors:** Xiaohui Xu, Keeley Brookes, Bo Sun, Nicholas Ilott, Philip Asherson

**Affiliations:** 1 MRC Social, Genetic and Developmental Psychiatry Centre, Institute of Psychiatry, King's College London, UK; 2 University of St Andrews, Bute Medical School, Fife, UK; 3 School of Medicine, King's College London, UK

## Abstract

**Background:**

Attention deficit hyperactivity disorder (ADHD) is a common, childhood-onset neurodevelopmental disorder that is more frequent in males than females. Several genes on the X chromosome have been studied as candidate risk factors for ADHD including the 5-HT_2C _receptor (HTR2C) gene. Association between polymorphisms in HTR2C and ADHD were reported in a recent study.

**Findings:**

In this study we investigated the association between ADHD and two polymorphisms C-759T (rs3813929) and G-697C (rs518147) in the promoter region of the HTR2C gene using a sample of 180 UK ADHD probands and their parents. We have shown that the -697G allele was significantly over-transmitted to affected ADHD probands (*P *= 0.017). No association was detected between the C-759T polymorphism and ADHD. Haplotype analysis of the two markers revealed no significantly increased transmission of any haplotype to ADHD.

**Conclusion:**

The findings provide evidence that the G-allele of the G-697C HTR2C polymorphism may be involved in the development of ADHD. The results replicate one of the findings published recently.

## Findings

Attention deficit hyperactivity disorder (ADHD) is a childhood onset, neurodevelopmental disorder characterized by inattention, hyperactivity and impulsivity. Molecular genetic and pharmacological studies suggest the involvement of the dopaminergic, serotonergic and noradrenergic neurotransmitter systems in the pathogenesis of ADHD. Polymorphic variants in several genes involved in regulation of the dopamine, and related neurotransmitter pathways are reported to be associated with ADHD [1-3].

Serotonin is a neurotransmitter in human brain, and involved in a variety of functions including learning, aggression and cognitive processes [4,5]. The serotonin receptors have been classified into at least seven families (5-HT1-7). The 5-HT_2C _receptor (HTR2C) gene is located on human chromosome Xq24. The previous studies showed that the 5-HT_2C _receptor is a key contributor to control of central dopamine functions [6,7] and may play an important role in the aetiology of mental disorders, including ADHD. ADHD is much more frequent in males than females [8,9]. Genes coding to the X chromosome have been suspected as candidates for ADHD [10-14].

Yuan et al. [15] have suggested that the single nucleotide substitution polymorphisms in the upstream region of the 5-HT_2C _receptor could be involved in the promoter activity, as the -759C and -697G allele had less promoter activity than the -759T and -697C allele. One study has investigated association between HTR2C and ADHD in the Han Chinese population [16]. Two polymorphisms of HTR2C were investigated in 488 ADHD families in the study: C-759T (rs3813929) and G-697C (rs518147). The results showed that the -759C allele, the -697G allele, and haplotype -759C/-697G were significantly over-transmitted to affected probands, while haplotypes -759C/-697C and -759T/-697C were under-transmitted to ADHD probands. Li et al. [16] also found that the -697G allele and haplotype -759C/-697G were significantly over-transmitted to ADHD combined type (ADHD-C) probands, and haplotype -759T/-697C was under-transmitted to ADHD-C probands. Another study conducted by Brookes et al. [17] analysed 51 genes in a European collaborative sample of 776 DSM-IV ADHD combined type cases collected by the International Multi-centre ADHD Genetics (IMAGE) project and found association signals in DRD4, DAT1 and 16 other genes. Twenty-three single-nucleotide polymorphisms (SNPs) in HTR2C gene were investigated in this study, however, no association was found in any of HTR2C SNPs including C-759T and G-697C (*P *= 0.891 and *P *= 0.613, respectively).

To provide further clarification of the reported association, in this study we examined the two associated polymorphisms previously reported in the Han Chinese population.

## Methods

### Sample

DNA was collected from 180 DSM-IV ADHD combined subtype probands, from both parents for 116 of the ADHD probands and from the mother alone for 64 of the probands. Cases were recruited from child behaviour clinics in South-East England and referred for assessment if they were thought by experienced clinicians to have a diagnosis of the combined subtype of ADHD under DSM-IV criteria, with no significant Axis I co-morbidity apart from oppositional defiant disorder (ODD) and conduct disorder (CD) and IQ greater than 70. Only those individuals fulfilling the recruitment criteria after completion of research assessments were included in the study. 96% of the sample was male. The age range was 5–15 years at the time of assessment (mean 10.41, SD 2.34). Parents were interviewed with a modified version of the Child and Adolescent Psychiatric Assessment (CAPA) [18]. Information on ADHD symptoms at school was obtained using the long form of the Conner's questionnaire [19]. The subjects gave their written informed consent, and this study was approved by the Ethical Committee of King's College London (Reference number: G9814668).

### Genotyping

The C-759T (rs3813929) and G-697C (rs518147) polymorphisms were genotyped using the method of PCR and enzyme digestion. Genomic DNA was amplified by using forward primers 5'-ATCTCCACCATGGGTCTCGC-3' (spanning nucleotide positions -885 to -866) and reverse primers 5'-CAATCTAGCCGCTCCAAAGG-3' (spanning nucleotide positions -653 to -634). The reaction was performed according to the protocol described in the previous study [15]. After denaturation at 95°C for 5 min, samples were amplified using 35 cycles of 96°C for 45 sec; 59°C for 30 sec; and 72°C for 30 sec, followed by a final step at 72°C for 10 min. The PCR products were incubated overnight at 37°C with *Aci*I (New England Biolabs) and the digested PCR products were run on 4% agarose gel containing ethidium bromide. Both the C to T substitution at nucleotide position -759 and the G to C substitution at position -697 abolish an *Aci *I sites; positions -726 and -645 are also fixed restriction sites for *Aci*I. For the C-759T polymorphism, the C allele is represented by a 126 bp fragment, and the T allele by a 160 bp fragment. For the G-697C polymorphism, the C allele is represented by a 53 bp fragment, and the G allele by a 81 bp fragment.

### Data Analyses

Family genotype data were analysed using the transmission disequilibrium test (TDT) implemented in UNPHASED program (TDTPHASE) [20], . Since HTR2C is X-linked gene, genotype data from fathers was only used in the analysis of female ADHD probands. Genotype information from both parents in female probands, and from mothers alone in male probands was used to analyse linkage disequilibrium (LD). The Bonferroni correction was applied for multiple tests and *P *< 0.025 was considered to show a statistically significant difference.

## Results and discussion

The allele frequency of the two markers was estimated from the probands' mothers. For C-759T polymorphism the C-allele frequency is 76% and the T-allele frequency is 24%. For G-697C polymorphism the frequency of G-allele and C-allele is 48% and 52%, respectively. TDT analysis [Table 1] showed that the -697G allele was significantly over-transmitted to affected probands (χ^2 ^= 5.69, *P *= 0.017, OR = 1.7). Even after correcting *P*- values using the Bonferroni method for multiple tests a significant association was still found between the -697G allele and ADHD. Nevertheless, this finding should be treated with caution until further replication studies have been completed, since the significance level falls far below that required to adjust for the number of independent genetic variants across the genome (requiring a significance value of around 5 × 10^-8^). No difference in the transmission of any alleles of the C-759T SNP to ADHD was found (*P *= 0.384). Moderate LD was observed between the HTR2C markers (D' = 0.63). Haplotype analysis of the two markers revealed no significantly increased transmission of any haplotype to ADHD (global χ^2 ^= 4.52, 3df, *P *= 0.210).

**Table 1:** TDT Analysis of HTR2C Polymorphisms

	C-759T		G-697C	
		
	Allele		Allele	
	C	T	G	C
Transmitted	36	29	54	32
Non-transmitted	29	36	32	54
χ^2^, df (*P*-value)	0.755, 1df (0.384)		5.69, 1df (0.017)	
OR	OR = 1.2		OR = 1.7	

Genes coding to the X chromosome have been suspected as candidates for ADHD because ADHD is much more frequent in males than females [8,9]. Previous studies have reported that the monoamine oxidase A gene (MAOA) located on the X chromosome was associated with ADHD [10-14]. The DXS7 marker, being on X chromosome and closely linked to MAO genes, has been investigated in ADHD [21,22]. Significant association and linkage were detected between the DXS7 polymorphism and ADHD [21]. However, this finding was not replicated in Irish ADHD samples [22]. To our knowledge, so far only two studies carried out association study between HTR2C gene located on X chromosome and ADHD [16,17]. Reference [16] investigated association between polymorphisms (C-759T and G-697C) in HTR2C gene and ADHD and found association between the two polymorphisms and ADHD in Han Chinese subjects. However, no association was found in the two SNPs in HTR2C gene with ADHD in a sample of 776 DSM-IV ADHD combined type cases [17].

In this study, we carried out further investigation between polymorphisms in HTR2C gene and ADHD in UK samples. We found a significant over-transmission of the -697G allele to ADHD combined subtype cases. Our study replicates a finding conducted by Li et al. [16], who found that a preferential transmission of the -697G allele to ADHD probands (*P *= 0.010) and to ADHD-C probands (*P *= 0.041). We did not observe any association with the C-759T polymorphism and ADHD in our samples, and was thus unable to replicate the finding for this SNP by Li et al [16]. Haplotype analysis of the two markers revealed no significantly increased transmission of any haplotype to UK ADHD cases.

## Conclusion

In this study we only observed that the -697G allele was associated with ADHD in a UK population. The finding suggested that the -697G allele may be a risk factor in the development of ADHD although this conclusion was not supported in a large multi-site European sample of ADHD [17]. Due to the small sample sizes in our study and relatively few published data on the HTR2C polymorphisms investigated in ADHD, further association studies are needed to confirm or refute the finding.

## Competing interests

The authors declare that they have no competing interests.

## Authors' contributions

XX selected the SNPs, performed genotyping, statistical analysis and drafted the manuscript. KB sorted out the samples. BS and NI participated in the genotyping for the study. PA supervised the study. All authors contributed to the final critical revision of the manuscript.
